# Confronting a Paradox: A New Perspective of the Impact of Uncertainty in Suspense

**DOI:** 10.3389/fpsyg.2018.01392

**Published:** 2018-08-08

**Authors:** Pablo Delatorre, Carlos León, Alberto Salguero, Manuel Palomo-Duarte, Pablo Gervás

**Affiliations:** ^1^Department of Computer Science, University of Cadiz, Cádiz, Spain; ^2^Department of Software Engineering and Artificial Intelligence, Instituto de Tecnología del Conocimiento, Universidad Complutense de Madrid, Madrid, Spain

**Keywords:** suspense, uncertainty, anticipation, cognitive model, computational creativity

## Abstract

Suspense is a key narrative issue in terms of emotional gratifications. Reactions in response to this type of entertainment are positively related to enjoyment, having a significant impact on the audience's immersion and suspension of disbelief. Related to computational modeling of this feature, some automatic storytelling systems include limited implementations of suspense management system in their core. In this way, the interest of this subject in the area of creativity has resorted to different definitions from fields as narratology and the film industry, as much as several proposals of its constituent features. Among their characteristics, uncertainty is one of the most discussed in terms of impact and need: while many authors affirm that uncertainty is essential to evoke suspense, others limit or reject its influence. Furthermore, the paradox of suspense reflects the problem of including uncertainty as a component required in suspense creation systems. Due to this need to contrast the effects of the uncertainty in order to compute a general model for automatic storytelling systems, we conducted an experiment measuring suspense experienced by a group of subjects that read a story. While a group of them were told the ending of the story in advance, the members of the other group experimented the same story in chronological order. Both the subjects' reported suspense and their physiological responses are gathered and analyzed. Results provide evidence to conclude that uncertainty affects the emotional response of readers, but independently and in a different form than suspense does. It will help to propose a model in which uncertainty is processed separately as management of the amount of knowledge about the outcome available to the spectator, which acts as a control signal to modulate the input features, but not directly in suspense computing.

## 1. Introduction

Suspense plays a key role in creating emotional immersion in narrative and keeping readers' attention through the story (Khrypko and Andreae, 2011, p. 5:1). Reactions in response to this type of entertainment are positively related to enjoyment (Oliver, 1993, p. 315), having a big impact on the audience's immersion and suspension of disbelief (Hsu et al., 2014, p. 1359). A study run by Schraw et al. (2001) concluded that readers find literary texts interesting when the content is suspenseful, coherent, and thematically complex, accounting for approximately 54% of the variance in situational interest, where suspense made the single greatest contribution, explaining roughly 34% of variation (p. 445). Thus, it is a technique broadly used by authors and storytellers in a variety of narrative domains, including objectives different beyond the classical entertainment^1^.

This evidence that suspense is a key factor in different areas and a wide range of narrative media motivates the strong interest of computational creativity in the field. Actually, a number of automatic storytelling systems try to produce suspense when creating plots. For this aim, not only a correct implementation of the generation system aiming to evoke suspense, but also modeling the concept in itself is required previously. This implies to adequately define what suspense is and, ultimately, to identify its constitutive components.

Nowadays the computational modeling of suspense represents a considerable challenge due to the fact that, despite the importance of the phenomenon, there is still no single unified theoretical definition of suspense. This is understandable given the difficulty that analyzing complex behavior implies. Suspense, being affected by a probably high number of measurable aspects of general cognition, is no exception. The literature on the subject lists a number of different proposals coming from narratology (Tan, 1995; Abbott, 2008; Branigan, 2013), psychology (Comisky and Bryant, 1982; Ortony et al., 1990; Gerrig and Bernardo, 1994), or entertainment theory (Zillmann, 1996; Frasca, 2003; Frome and Smuts, 2004).

While these definitions share –sometimes implicitly– a number of features –for instance, outcome transcendence, fear and, ultimately, anticipation–, in other cases there is no agreement on their impact on suspense even though there are significant contributors to it. Among the controversial characteristics are moral balance, the effect of audience memory, empathy with characters and –probably the most discussed aspect– uncertainty about the outcome. As detailed in section 2, the influence of uncertainty in suspense is a “hot spot.” On the one hand, it could seem that suspense is not possible when the audience knows what is going to happen. On the other hand, the inclusion of uncertainty as constitutive part of suspense leads to a paradox: if suspense requires uncertainty, it would not be possible to feel suspense the second time that we experience a film. For decades, several attempts have been made to explain this question, but none of the theories is free of criticism and possible inconsistencies. Thus, nowadays the paradox is still a matter of discussion, and the role of uncertainty in suspense is still unclear. Consequently and as described in section 2.3, current automatic storytelling systems focused on suspense rarely consider uncertainty explicitly in their architectures.

For example, in Delatorre et al. (2017) we propose the architecture of a system whose main objective is the adaptation of the descriptive elements of a suspenseful scene, in such a way that the amount of information of the scene output is adjusted to the required suspense intensity. The system manages the structural components of the scene based on a *weighted corpus* consisting of a set of concepts, each one associated with a quantitative value that represents its level of suspense. As most of current automatic generators of stories, the system is still lacking the explicit treatment of uncertainty. With the aim to add this feature, firstly we need to clarify the impact of uncertainty on the perception of suspense.

Based on the introduced background, the current research tries to advance toward a formalization of the suspense as a cognitive phenomenon. The main objective is to shed some light on the possibilities of the computational-cognitive modeling of such a complex subject, concretely in terms of whether uncertainty should be a constitutive component or not.

As previously introduced, the fact that suspense can be experienced even when no uncertainty is possible has led us to believe that suspense and uncertainty are independent feelings. They can appear simultaneously, but we defend that they must be studied separately. The main hypothesis guiding the study can be summarized as follows:

Emotions experienced by the audience in a suspenseful scene involve –although it is not limited to– an optional component called ‘uncertainty’ that is generated by a lack of information and it is not restricted to suspenseful scenes. This component is different from the mandatory component called ‘suspense,’ that is generated by an outcome anticipation.

According to this hypothesis, we support that uncertainty is not a constitutive component of suspense. In order to support this, we conducted an experiment in which sixty eight subjects individually read a version of a short story two consecutive times, reporting the level of suspense that they felt for each one of the twelve passages in which the story was divided. Responses were analyzed to check if uncertainty influences reported suspense, in addition to other variables as the style or the ending of the story.

The main aim of this contribution is to provide evidence that suspense may exist independently of uncertainty and, ultimately, uncertainty is not a feature of suspense. Exploring the effects of this hypothesis is a relevant objective for automatic storytelling.

Beyond their utility to create a suspenseful story, other aspects influencing it —outcome, empathy, environment— are not directly studied.

The rest of the paper is organized as follows: section 2 describes the related literature on suspense and uncertainty. section 3 explains the experiment, whose results are analyzed in section 4 and discussed in section 5. Finally, section 6 summarizes the overall contribution.

## 2. Related work

In this section we review different approaches to uncertainty is suspense and several automatic storytelling systems focused on suspenseful story generation.

### 2.1. Discussions about uncertainty as factor of suspense

Uncertainty refers to the state of an organism that lacks information about whether, where, when, how, or why an event has occurred or will occur (Knight, 1921). It has been defined as a lack of information about an event and has been characterized as an aversive state that people are motivated to reduce (Bar-Anan et al., 2009, p. 123) or –less often– prolong in the case of following a positive event (Wilson et al., 2005, p. 5). It is related to a state of curiosity in which people desire more information about something that produces pleasure only when it is satisfied (Lowenstein, 2011, p. 75).

For its part, suspense is broadly described as an effect of anticipation (Nomikos et al., 1968; de Beaugrande, 1982; Carroll, 1990; de Wied, 1995; Mikos, 1996; Wulff, 1996; Yanal, 1996; Prieto-Pablos, 1998; Vorderer and Knobloch, 2000; Caplin and Leahy, 2001; Allen, 2007; O'Neill, 2013). In a suspenseful passage, the reader expects or anticipates the outcome of the protagonist (Iwata, 2009, p. 30), and this state remains until the presentation of the outcome event (de Wied, 1995, p. 111). According to this, specific definitions and components of suspense vary according to the authors' point of view.

The existing academic literature provides several definitions that discuss the influence of uncertainty in suspense. Ortony et al. (1990, p. 131) affirm that, along with fear and hope, a “cognitive state of uncertainty” is one of the three component of suspense. For Zillmann (1991, p. 283), suspense is conceptualized as the “experience of uncertainty regarding the outcome of a potentially hostile confrontation.” Perron (2004, p. 134) defends that the notion of uncertainty is, “without a doubt,” at the core of suspense: when a danger or threat is revealed and you are sure of the situation's outcome, there is no suspense. Iwata (2009, p. 36) points a relation between increasing reader's uncertainty and inducing suspense. Madrigal et al. (2011, p. 261) also relate that uncertainty over how an episode will end is a core ingredient of suspense. Likewise, Knight and McKnight (1999, p. 108) claim that “suspense relies upon the audience's strong sense of uncertainty about how events will play out.” For Khrypko and Andreae (2011, p. 5:2), the key element in suspense is uncertainty about which of the possible outcomes is going to occur when there is a balance between desired and non-desired outcomes. Along with this, O'Neill (2013, p. 9) affirms that the degree of suspense is correlated with the reader's uncertainty over the means of escape for a hero. Abbott (2008, p. 242) defines suspense as “uncertainty (together with the desire to diminish it) about how the story will develop,” linking its resolution with some degree of surprise. A similar definition is proposed by Carroll (1996b, p. 101), who alleges that classical suspense implies to question about “what happens next?” Analogously, Frome and Smuts (2004, p. 16) assure that, while suspense depends on something at stake, if there is no uncertainty, then can be no suspense. Lauteren (2002, p. 219) affirms that “the element of suspense is created through the uncertainty of its outcome.” For Wulff (1996, p. 7), suspense comes from the uncertainty –about character's roles, intentions, etc, what he includes in the concept of *anticipation*–, as the degree of probability with which the story can develop in one or another direction can be calculated. Prieto-Pablos (1998, p. 100) describes a type of suspense which is the consequence of our cognitive response to conditions of uncertainty.

All these definitions claim that suspense requires uncertainty about a particular outcome –on the part of the audience–, where the outcome is significantly desirable or undesirable (O'Neill and Riedl, 2014, p. 944).

Controversially, some authors question uncertainty as a factor implied in suspense. Hitchcock himself implicitly questioned this feature, observing that the key feature of suspense is that the audience be aware of the anticipated outcome: “in the usual form of suspense it is indispensable that the public be made perfectly aware of all of the facts involved” (Truffaut, 1985, p. 72). This is supported by other affirmations. Burget (2014, p. 45) affirms that suspense is a fear emotion about an outcome, and the spectators can fear the outcome of a situation despite the fact that they know it. Smuts (2008, p. 284) links uncertainty with surprise, and claims that “surprise is clearly not involved in all or even most cases of suspense.” Moreover and based on an example of Walton (1978) in which a kid felt repeated suspense even when he had memorized all the story, Gerrig (1997, p. 168) also questions the role of uncertainty. Like him, other authors who consider uncertainty as part of suspense also cast doubts about its level of influence. For instance, (Hoeken and van Vliet, 2000) do not appear to take uncertainty of the story's outcome to be so vital to suspense creation, considering other narrative techniques more significant (Iwata, 2009, p. 29). Zillmann (1996, p. 102) points that uncertainty is not introduced by explicit description, but rather introduced implicitly by the suggestion of a number of possible negative outcomes. That could mean that, taken together with the fact that suspense is not maximized when the uncertainty is maximized^2^ (van Vught and Schott, 2012, p. 95), uncertainty could be overvalued as a factor in the production of suspense (O'Neill, 2013, p. 10). In the same vein, Frome and Smuts (2004, p. 17) add that “higher uncertainty might make the scene more suspenseful, but if enough is at stake, you can have suspense even with a likely desirable outcome.” Ryan (2001, p. 180) defends that suspense requires, in addition to the empathy with the character, that the audience perceives different potential outcomes of the situation even though there will be uncertainty about which of the outcomes will occur; however, the more potential outcomes the situation presents, the weaker suspense is. Oliver and Sanders (2004, p. 251) defend that suspense is related with the impression that the protagonist's suffering is very likely –uncertainty is involved but at a low degree– and the film that they are considering ultimately shows the protagonist escaping. Additionally, de Wied (1995, p. 113) proposes that suspense may be more intense the higher the viewer's subjective certainty about when in time the outcome event will occur –keeping viewers for one or two seconds in a heightened state of uncertainty may add to suspense–, except in instances of total subjective certainty. He defined the experience of suspense as an anticipatory stress reaction, prompted by an initiating event in the discourse structure, and terminated by the actual presentation of the harmful outcome event, focusing the duration of this anticipation as essential factor (p. 111).

None of the experimental approaches to the matter seem to resolve the controversy entirely. For instance, in the experiment of Comisky and Bryant (1982) a “balanced collision” could be expected between both uncertainty and suspense, but, instead, low levels of perceived outcome-uncertainty to produce high levels of suspense to certain point, in which suspense seems to decrease to its lowest value (Comisky and Bryant, 1982, p. 57). Comparable results were obtained by Epstein and Roupenian (1970), who found that a probability of 5% or less evokes the highest psychological responses. Along with these authors, Zillmann (1996, p. 208) defends that suspense increases as the uncertainty decreases a minimum just before total certainty. Likewise, (Iwata, 2009) experiments for creating suspense and surprise in short literary fiction conclude that, for a narrative episode to be suspenseful, a state of uncertainty must be sustained for a certain period —or space— in the story, whereas the duration of sustainment is not easily definable in a measurable way (Iwata, 2009, p. 136, 139). However, the author comes to define “uncertainty” as “delay in showing the resolution” (p. 174), which can differ from the notion of *unawareness* used by other studies. Nevertheless, (Niemelä, 1969) and (Breznitz, 2013) detected an increasing of heart rate as the probability of success increased. A third group —the experiment of Cantor et al. (1984) and (Monat et al., 1972)— shows that knowledge about an upcoming frightening event does not affect the “emotional defenses” of the audience. Conversely, subjects who were being warned reported a higher fright that those who were not warned, although anxiety seemed not affected by this forewarning (Cantor et al., 1984, p. 23, 30). Likewise, in a study entitled “Suspense is the Absence of Uncertainty” based on non-fictional texts, Gerrig (1989, p. 645, 646) defends that suspense does not need uncertainty, but it arises because audience repeatedly immersed in an episode —although repeated— would fail to seek out appropriate information in long-term memory. Based in a similar experiment, Hoeken and van Vliet (2000, p. 284, 286) conclude that, apparently, uncertainty about a story's outcome is not a prerequisite for the story to be suspenseful and, consequently, suspense is not simply the result of uncertainty about the outcome.

Summing up, the relationship between the degree of perceived uncertainty about the outcome and the amount of experienced suspense is not clear (Comisky and Bryant, 1982, p. 51). Current theories of suspense do not include a robust account for varying probability, and there is no consensus on the relation between probability and uncertainty (Guidry, 2004, p. 131).

### 2.2. The paradox of suspense

In addition to this debate, the inclusion of uncertainty as factor of suspense leads to an apparent inconsistency. Yanal (1996, p. 148) enunciated the concept of the paradox of suspense to describe this fact. The inconsistency is patent when observing the reactions of spectators exposed to a narrative more than once, referred to as *repeaters* (p. 147; Gerrig, 1997, p. 168).

In brief, the paradox of suspense can be explained like this: (i) repeaters experience suspense regarding a certain narrative's outcome; (ii) repeaters are certain of what that outcome is; (iii) suspense requires uncertainty. Further, these points were re-written and classified as following (Uidhir, 2011a, p. 122): (i) suspense requires uncertainty –*Uncertainty Premise*–; (ii) knowledge of a story's outcome precludes uncertainty –*Knowledge Preclusion Premise*–; (iii) we feel suspense in response to some narratives even when we have knowledge of the outcome –*Repeater Suspense Premise*–. In words of Smuts (2008, p. 282): “If uncertainty is integral to the creation of suspense, then how is it that some films can still be suspenseful on repeated viewings?”

Directly or not, so far there have been many attempts to solve this paradox, either from —mainly— theoretical (Brewer, 1996; Carroll, 1996a; Prieto-Pablos, 1998; Smuts, 2009; Uidhir, 2011b; Manresa, 2016) and experimental perspective (Comisky and Bryant, 1982; Iwata, 2009; Klimmt et al., 2009; Ian, 2012), analyzing the real impact of the uncertainty in suspense. In all cases, to resolve the paradox of suspense, the two workable options are proposed (Uidhir, 2011b, p. 163): deny the necessity of uncertainty or deny repeater suspense. Although some of these points have been exposed above, the main theories are summarized next.

Firstly, (Yanal, 1996) denies the existence of the paradox by rejecting repeater suspense. In this way, seeing a potential suspenseful scene again does not evoke “the same as feeling suspense,” but “a certain quality perhaps easily misidentified as suspense, namely anticipation” (Yanal, 1996, p. 157). On the basis that uncertainty is required for suspense, Yanal argues that, if true repeaters experience any kind of emotional response to suspenseful situation, their emotions must be of another kind (Prieto-Pablos, 1998, p. 109). Thus, he classifies re-readers who seem to be experiencing suspense into one of two categories: either they have forgotten some aspects of the story —in which case they are not really repeaters, so he also rejects the paradox explanation of Gerrig (1989)—, or, as said, what they are experiencing is some combination of other emotions –such as aforementioned anticipation–, which do not require uncertainty (Ian, 2012, p. 14). Yanal does not deny that repeaters experience emotions with respect to narratives, only that they do not experience suspense (Gerrig, 1997, p. 170) or at least not any emotion “grounded in uncertainty” (Yanal, 1996, p. 157).

On the other hand, it seems clear that recipients can reuse media not only to re-experience the same emotions, but also due to any other motivations and gratification research (Hoffmann, 2006, p. 393). However, there is not enough evidence that it prevents the audiences from having the same or near the same experience (Burget, 2014, p. 46), which would contradict Yanal's discourse.

On his part, Gerrig differs from Yanal in the sense that he considers that the audience revive some kind of internal representation and reaction, and this would argue strongly that Yanal is wrong. Gerrig reuses Yanal's own example of Marion Crane in the shower, in the film *Psycho* (Stefano, 1959). Gerrig describes that some subset of repeaters would hear their mental voices call out “Get out of the shower!” or “Look out!,” which would reflect momentary uncertainty that what the repeaters *know* to happen does, in fact, happen. Willing to take these mental voices as evidence, Gerrig see that knowledge of the outcome produces more rather than less moment-by-moment uncertainty (Gerrig, 1997, p. 171). Accordingly, Gerrig rejects the paradox, affirming it is the result of what he calls “anomalous suspense,” what makes repeaters genuinely re-experience suspense (p. 168). For him, it is an emergent property of ordinary memory processes, suggesting that memory processes actuate an *expectation of uniqueness* (p. 172), reflecting “a systematic failure of memory processes to produce relevant knowledge as a narrative unfolds” (p. 172). That is, repeaters are not really repeaters in the strict, operative sense, but instead more loosely akin to narratively functional amnesiacs —or operatively offline repeaters— (Uidhir, 2011b, p. 162). Consequently, audience expects a unique outcome regardless of the circumstances in which it finds itself respect to the narrative events (Prieto-Pablos, 1998, p. 110).

However, Uidhir (2011b, p. 162) mentions that Gerrig could fall into some imprecisions. On the one hand, he claims that “in some (but not all cases) and given certain conditions and dispositions” —which is clearly inaccurate—, repeaters when narratively engaged can be sufficiently immersed in or transported by the narrative so as to render their experiences saliently approximate to non-repeater experiences. On the other hand, it may be argued that Gerrig employs far too broad a notion of repeater and so merely substitutes one imprecision for another. Likewise, Carroll (1996a, p. 90) argues that, if re-reading implied *uniqueness*, it would not be possible to get bored even after a number of repeated similar experiences.

Carroll proposes an extended theory of suspense, in which suspense is an emotional response to narrative fiction which requires not just uncertainty but also moral concern for the outcome, an emotion which he suggests readers continue to feel even in the absence of uncertainty (Ian, 2012, p. 14). To solve the paradox, Carroll distinguishes real beliefs from fictional beliefs, in which thoughts about them can give rise to emotions as suspense. Thus, effectively asked to imagine —that is, to entertain the thought— that the main character is at risk by the author of the fiction, the audience appropriately and intelligibly feels concern and suspense (Carroll, 1996a, p. 90). He argues that even if we know that a film will end in a certain way, we can still imagine, while watching it, that it could not end that way. Merely imagining that an event's outcome is uncertain is enough to create suspense (Frome and Smuts, 2004, p. 19).

From this point of view, Carroll rejects the *Knowledge Preclusion Premise* –that is, knowledge of a story's outcome precludes uncertainty–, due to the audience may be “entertaining the mind” with fictional alternatives. Nevertheless, he does not get to explain how the psychological mechanism works to get a new mental state of uncertainty, remaining his contribution in an uncompleted and merely theoretical plane (Manresa, 2016, p. 58). Moreover, Ohler and Nieding (1996, p. 139) question the morality as the basis on suspense, considering that a moral concern evoked by a scene is not necessarily a prerequisite to experience suspense.

Unlike the previous authors, in his *desire-frustration theory* Smuts (2008, p. 284) rejects the most widely accepted premise of the paradox –the *Uncertainty Premise*, or the assumption that suspense requires uncertainty–. Instead, he affirms that suspense is felt by the frustrated desire to “jump” in the scene and to “help” the characters: “Our desire to make use of the information is frustrated –that is, we want to help, but there is nothing we can do–” (p. 285). Thus, suspense lays on the basis of manipulating the narrative information to create emotional situations when the audience is “forced to entertain the prospect of a narrative outcome which is contrary to the one that is desired” (Allen, 2007, p. 38) and the frustration comes from the inability to influence narrative (Burget, 2014, p. 49). As uncertainty is not necessary, this indeed would solve the paradox.

However, Smuts himself objects to his own theory when applied to real cases –as a lottery–, in which he affirms that, in this cases, uncertainty is essential for suspense. Actually, he notes that “uncertainty is not necessary for *all* cases of suspense, that one can feel suspense on *some occasions* without uncertainty” (Smuts, 2008, p. 287).

On the basis of the above, other proposals have been made. For example, Manresa (2016, p. 63) explains the repeated suspense as the result of a process of re-sympathizing with the characters, and Prieto-Pablos (1998, p. 111) affirms that the paradox can be explained by taking into consideration the potential variability of the different emotions that are involved in a narrative experience.

In the words of Beecher (2007, p. 258), all proposals to explain the paradox “are ingenious but not entirely convincing.” Actually, none of these proposals is entirely free of possible inconsistencies. Thus, the paradox of suspense remains as a matter not resolved yet.

### 2.3. Uncertainty in automatic storytelling

This section summarizes how automatic story generation systems address uncertainty.

MEXICA (Pérez y Pérez, 2007) is a program that generates short stories about the old inhabitants of what today is Mexico City (p. 2). These stories are represented as clusters of emotional links and tensions between characters, progressing during development. MEXICA assumes that a story is interesting when it includes degradation-improvement processes –i.e., conflict and resolution– (p. 4). Throughout the history, emotional links among the characters vary as a result of their interactions. However, uncertainty is not explicitly treated in the system.

MINSTREL (Turner, 2014) is a complex program that writes short stories about Arthurian legends, implemented on a case-based problem-solver where past cases are stored in an episodic memory (Pérez y Pérez and Sharples, 2004, p. 4). MINSTREL recognizes narrative tension plots and tries to increase the suspense by adding more emotionally charged scenes, by storing a simple ranking which tells when such inclusion is reasonable (Turner, 2014, p. 123). Just like in MEXICA, there is no specific implementation of uncertainty.

IDtension (Szilas, 2003) is a drama project which comes up to demonstrate the possibility of combining narrative and interactivity. Unlike approaches based in character's chances or the course of the actions, it conceives the stories based on narrative properties –conflict or suspense–. It neither include uncertainty as explicit part of the computational model, but as part of a general manage of “known” information (p. 768).

Another initiative is Suspenser (Cheong and Young, 2006), that creates stories with the objective of increasing the reader's suspense. It provides an intermediate layer between the *fabula* generation and the discourse generation, which selects the steps of the plot according to their *value of importance* for the final goal. For this and based on the assumption of Gerrig and Bernardo (1994)^3^, Suspenser uses a set of heuristics grounded in the number of paths available for the character to reach its goal, considering optimal the probability of protagonists' success as 1/100 (Cheong, 2007, p. 59). To meet the uncertainty condition of suspense, the suspense measurer first checks if the reader model would be uncertain about the goal state using the planning space^4^. Therefore, the model returns certainty when the planning space contains either only complete plans –absolute success– or only failed plans –absolute failure– (Cheong and Young, 2015, p. 44).

Also based in Gerrig & Bernardo's work, Dramatis (O'Neill, 2013, p. 5) proposes an implementation of a system to evaluate suspense in stories that utilizes a memory model and a goal selection process, assuming that the reader, when faced with a narrative, evaluates the set of possible future states in order to find the best option for the protagonist, which seems to assume the treatment of uncertainty. Actually, the requisite state of uncertainty is implicitly represented as the reduction in possible escapes for the protagonist from the negative outcome (p. 31).

In summary, only one of analyzed systems offers an explicit computational treatment of uncertainty. A possible explanation is that the execution of the plan in the absence of uncertainty guarantees that the goal state will become true, which allows for a better control of the results. The only reason why the planner would have any uncertainty as to the true state of the story world would be if the human author specified that there was uncertainty (Riedl, 2004, p. 48, 120). In any case, we cannot rule out another reason as that there is no general agreement on the impact of the uncertainty on suspense, as we have recounted.

## 3. Experiment

The present study was carried out in accordance with the recommendations of national and international ethics guidelines, *Código Deontológico del Psicólogo* and American Psychological Association. The study does not present any invasive procedure, and it does not carry any risk to the participants' mental or physical health, thus not requiring ethics approval according to the Spanish law BOE 14/2007. All subjects participated voluntarily and gave written informed consent in accordance with the Declaration of Helsinki. They were free to leave the experiment at any time.

The experiment was carried out to support the aforementioned hypothesis that emotions experienced by the audience in a suspenseful scene involve —although it is not limited to— an non-specific optional component called “uncertainty” that is generated by a lack of information, and which is different from the mandatory component called “suspense” that is generated by outcome anticipation. In order to do that, sixty eight (68) subjects were asked to read a version of a short story two consecutive times, reporting the level of suspense that they feel for each one of the twelve passages in which the story is divided. Responses were analyzed to check if uncertainty influences reported suspense, in addition to other variables as the style or the ending of the story.

This section describes the methodology and the experiment. Section 4 describes the corresponding results.

### 3.1. Material

For conducting the experiment, a suspenseful short story was written under the guidance of a professional writer. With the aim of evaluating the effect of the uncertainty separately from other potential emotions, it was grounded under the following premises.

Firstly, a person had to be faced with a crucial outcome. In order to maximize the suspense of the story, a child was chosen as this victim^5^. Likewise, the outcome is dying. Actually, about to die is the “initial state” of the victim –waiting in a hospital for an urgent transplant–, with the possibility of being saved if the organ –a liver– reaches the hospital in time. To avoid interferences with this core, no other victim or threat were included in the story.

Secondly, to limit the potential resources applicable by the reader to infer possible measures for the victim to –consciously and/or directly– counteract the threat, the story included a first paragraph telling that it narrates a real case. With this strategy, the result of the story depends on the arrival of the liver. Other choices –anything that induces that a “miracle” is possible– from science fiction, fantasy, any other fiction genre or even a true history plot when a not-known element is introduced in the story (Gerrig, 1989, p. 633) are discarded. Thus, it restricts the readers' hope for the victim to be saved “in the last minute” even if the organ is not in time. Actually, a surgery in a hospital was chosen on the basis of the story to recreate a more realistic and fictionally restricted environment, which would not be such easier by referencing other suspenseful classical scene, involving dark alleys, and killers or “scary-looking monsters” (van Vught and Schott, 2012, p. 100).

**Figure 1:**
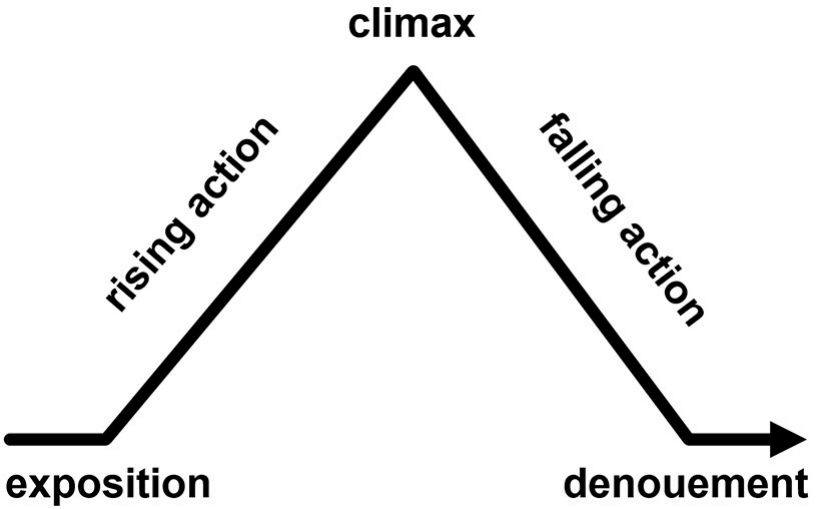
Freytag's pyramid.

Thirdly, the plot follows the classical pyramidal scheme proposed by Freytag (1894), as presented in Figure 1, as detailed: In *exposition* stage, context, victim state and chances are introduced –two men transport the liver along the hospital–; later, in *rising action* stage, the text prepares the readers for a potential incoming obstacle –the floor is wet–; in the *climax*, the situation becomes seriously complicated to counteract the threat –the man who carries the liver slides, and the case in which this is transported seems broken–; a *falling action* stage resolves the plot –medical team study the liver's conditions–; finally, in order to test if the final outcome influences suspense once the ending is known, the story presents two possible endings at the *denouement* stage concerning the valence: (a) *good ending*, in which the liver is still suitable for the transplant and the child survives; and (b) *bad ending*, in which the liver has not survived the impact and the child dies some hours later. The story was composed in twelve passages, each one containing one to three sentences. Structurally, it is conceived as a short story structured as follows: *exposition*, from passage 1 to 3; *rising action*, from 4 to 8; *climax*, 9 and 10; *falling action*, 11; and, *denouement*, passage 12.

Additionally, to control the effect of how the story is written, two versions were developed: (a) a version made of short sentences and a journalistic style; (b) a version using a short novel style. Furthermore, the ending is introduced either: (a) only in the last passage; or (b) in the first passage too, with the aim to test the effect of the previous knowledge of the outcome in the first exposition. In order to avoid a potential emotional influence due to the way in which this ending is presented, the narrative style of the first and the last excerpt was the same both in the short novel and the journalistic version.

In this way, different versions of the story were created with respect to the features: revelation of the ending —in the first excerpt—, valence of the ending —good or bad— and style —journalistic short style or novel long style—. To create the plots, characters and the discourse, we were assisted by the Spanish novelist Rafael Marín^6^. The set of resulting stories is provided in the appendix “Story” (see Supplementary Material).

### 3.2. Participants

The experiment was announced and those wanting to take part in it voluntary enrolled, counting finally sixty eight undergraduate students (*N* = 68), 40 males (59.82%) and 28 females (41.18%), from the University of Cadiz (Spain), with ages ranging from 17 and 32 years (*mean* = 21.14, *stdev* = 2.98). All participants were Spanish native speakers. There was no compensation for participating in the experiment.

Each participant was assigned an internal code –from 01 to 68–, relating this code with age, genre and contact method. By means of this code, participants were anonymously assigned to story types, to ensure a balanced distribution of participant —number of participants, age and genre— over the features to be tested in stories –revelation of ending, valence and style, as explained–.

This distribution was manually carried out, for which the set of participant was ordered by genre and age. Table 1 shows the distribution of the participants.

**Table 1:** Experiment participant's distribution.

**Gender**	***N***	**Ratio**	***Mean*_*age*_**	***SD*_*age*_**
*Global*	68		21.14	2.98
Male	40	58.82%	20.53	2.28
Female	28	41.18%	22.03	3.63

The experiment was conducted from March to June, 2017.

### 3.3. Method

Participants were individually called for the experiment. Each participant was taken to a seminar room. After a general explanation of the process, the participant filled a demographic survey. In the first page the participant was informed that anonymized data would be collected and that going on with the evaluation implied the acceptance of these conditions. All participants agreed.

Participants start reading the story in a 19″ monitor laptop. Garamond, 24pt, was the chosen font. Each story was divided in twelve slides, as many as passages.

Revelation of ending —in the first slide—, valence of the ending —good or bad— and style —journalistic or novel— were blindly and equally distributed among the participants, as explained in section 3.2. The subjects were previously required to report the level of suspense they felt after reading each slide. For this, a 9-point rating scale was used as a paper-and-pencil survey, where scale was presented as a pictographic scale based on the SAM model (Bradley and Lang, 1994). Immediately after a first run, each subject was given a new paper with another group of 9-point SAM model and was required to read all the slides again, also annotating, for each slide, the level of suspense.

## 4. Results

After running the experiment, demographic information for all participants and a total of 1632 report lines were collected. Each of these report lines included the reported suspense for each passage and story features. Three (3) entries had to be discarded because they were missing some parts or erroneous.

**Figure 2:**
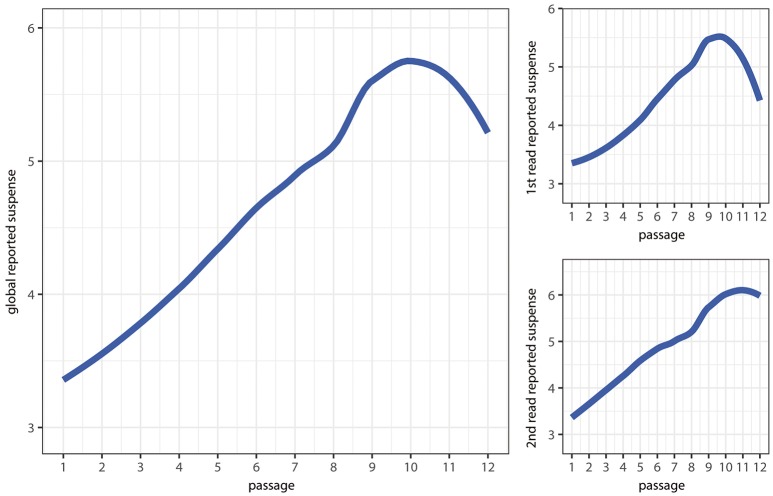
Suspense curves through story passages.

Figure 2 represents reported suspense through the twelve passages of the story. As expected, suspense increased during the plot until it raised the climax. After that, arousal descended in the last passages. When comparing first read with second read, it may be observed that suspense was higher in the second read; differences are remarkable as end is coming, which may be observed in Table 2. Additionally, climax and denouement stage seemed less pronounced in the second read. This fact and other results described below will be discussed in section 5.

**Table 2:** Reported suspense by passages and reading order.

		**First reading**		**Second reading**	

**Passage**	***Z***	**Mean (std)**	**Median**	**Mean (std)**	**Median**
1	0.684	3.32 (2.12)	3.0	3.36 (1.76)	3.0
2	1.327	3.53 (1.62)	4.0	3.92 (1.87)	4.0
3	−0.294	3.76 (1.75)	4.0	3.65 (1.98)	4.0
4	1.560	3.61 (1.94)	4.0	4.17 (2.09)	4.0
5	0.677	3.94 (2.24)	3.0	4.22 (2.32)	4.0
6	1.314	4.85 (1.79)	5.0	5.19 (1.94)	5.0
7	0.857	4.72 (1.91)	5.0	5.03 (2.29)	5.0
8	−0.524	4.94 (2.09)	5.0	4.68 (2.41)	5.0
9	1.764^*^	5.57 (1.99)	5.0	6.11 (2.24)	6.0
10	1.537	5.57 (1.76)	5.0	5.97 (2.19)	6.0
11	3.328^***^	5.91 (2.01)	6.0	6.97 (2.05)	7.5
12	3.802^***^	3.94 (1.71)	4.0	5.43 (2.25)	5.0

*** p-value < 0.001,

* *p-value < 0.05*.

A multiple analysis of variance (MANOVA) was conducted to provide evidences about the impact of each variable and their combinations^7^ as showed in Table 3.

**Table 3:** Influencing variables in reported suspense.

***F***	**Influencer**	**Mean (std)**	**Median**
*F*_(1, 1625)_ = 21.751^***^	*(ro) reading order*
	First	4.46 (2.00)	4.0
	Second	4.91 (2.24)	5.0
*F*_(1, 1625)_ = 16.676^***^	*(en) ending*	
	Good	4.90 (2.25)	5.0
	Bad	4.47 (2.21)	5.0
*F*_(1, 813)_ = 26.722^***^	*(kn) ending knowledge*	*(1st read)*
	Yes	4.12 (2.01)	4.0
	No	4.79 (1.97)	5.0
*F*_(1, 1625)_ = 1.915	*(st) style*	
	Long	4.76 (2.16)	5.0
	Short	4.61 (2.31)	5.0
*F*_(3, 1625)_ = 19.210^***^	*st / en*
	Long/good	4.76 (2.15)	5.0
	Long/bad	4.77 (2.17)	5.0
	Short/good	5.03 (2.35)	5.0
	Short/bad	4.21 (2.20)	4.0
*F*_(3, 813)_ = 74.695^***^	*st / kn (1st read)*
	Long/yes	4.01 (2.16)	4.0
	Long/no	5.95 (1.64)	6.0
	Short/yes	4.23 (2.01)	4.0
	Short/no	3.81 (1.84)	4.0
*F*_(3, 813)_ = 7.525^**^	*en / kn (1st read)*
	Good/yes	3.89 (2.05)	4.0
	Good/no	4.99 (1.93)	5.0
	Bad/yes	4.40 (2.09)	5.0
	Bad/no	4.67 (2.13)	5.0

*** p-value < 0.001,

** *p-value < 0.01*.

A deeper analysis derived from these results reveals some interesting points. Firstly and according to the variable *ending* (*en*), in general to anticipate surviving provoked more suspense than in the case of the death. It is not significant in the first reading, but it is clear when comparing reports in the second reading, as may be observed in Table 4. As trivially expected, ending was not a significant variable when audience do not know the outcome (*Z* = −0.374, *p* = 0.708), which happens only in first reading.

**Table 4:** Reported suspense by ending and reading order.

		**Good ending**		**Bad ending**	
**Reading**	***Z***	**Mean (std)**	**Median**	**Mean (std)**	**Median**
**Reading order**					
First	1.677	3.92 (1.96)	4.0	4.34 (2.04)	4.5
Second	−4.252^***^	5.37 (2.20)	6.0	4.45 (2.19)	4.0

*** *p-value < 0.001*.

Secondly, despite the fact that the story *style* (*st*) did not appear to affect reported suspense by itself, it is influential when the order of reading and the factor of knowing the ending are studied separately. As shown in Table 5, in the case of not knowing the ending, the long version appeared to provoke more suspense. However, a higher suspense is reported by the short adaptation in the case of the knowledge of the outcome. As pointed, the long version got a higher score in the first round, but only when the audience had not been previously notified about the outcome (*Z* = 10.509, *p* < 0.000). When the audience had been notified of the outcome previously, differences were lower, suspense being slightly higher in the short version (*Z* = −3.677, *p* = 0.001). This suggests that the long version provoked more suspense in the presence of uncertainty, while the short version provoked more suspense when the audience had been notified of the outcome in advance. It was also noticeable that a substantially higher suspense was experienced in all cases in which the audience had already read any version of the story before.

**Table 5:** Reported suspense by styles, ending knowledge and reading order.

		**Long version**		**Short version**	
**Variable**	***Z***	**Mean (std)**	**Median**	**Mean (std)**	**Median**
**Ending knowledge**					
Known	−3.677^***^	4.39 (2.17)	4.0	4.89 (2.39)	5.0
Unknown	10.509^***^	5.95 (1.64)	6.0	3.81 (1.84)	4.0
**Reading order**					
First reading	6.704^***^	4.98 (2.15)	5.0	4.02 (1.93)	4.0
Second reading	−4.223^***^	4.56 (2.15)	5.0	5.23 (2.51)	6.0

*** *p-value < 0.001*.

Finally, focusing on uncertainty Table 3 shows that, in the first reading, it influenced suspense [*F*(1, 813) = 26.722, *p* < 0.000]. However, it also reveals that the second reading evoked more suspense than the first one [*F*(1, 1625) = 21.751, *p* < 0.000].

No other significant dependencies between variables were found. Likewise, general and segmented reported suspense were not practically influenced by participants age (*r* = −0.169, *p* < 0.000) nor gender (*Z* = −0.974, *p* = 0.330).

## 5. Discussion

The observations obtained from the experiment reveal that: (a) uncertainty positively affected reported suspense in first reading; (b) however, while progression curves were similar although it is lower in the first reading—, the same stories were reported as more suspenseful in the second read; and (c) the existence of uncertainty provoked a higher scored suspense than the long version, while outcome knowledge implied a higher prevalence of suspense in the short version.

While the first observation may support the impact of interactivity in suspense, the second and third observations question it, which might seem to be a new paradox. We consider two possible explanations to this effect.

The first explanation would be that uncertainty is effectively a part of suspense –observation (a)– but it is not essential to create suspense –observations (b) and (c)–. Despite of the fact that this would solve the paradox, it must be clearly seen that, in the absence of uncertainty, the impact of other features of the story increases suspense in a repeated exposition. The accumulated effect of these factors can sometimes become emotionally stronger than the effect that of uncertainty. From this point of view, uncertainty as part of suspense would only contribute to the emotion experienced the first time in which the story is encountered. In subsequent expositions to the same story, other factors involved in suspense would take over in driving the experience of suspense.

An alternative approach is to deny that uncertainty is a feature of suspense. Such an approach suggests that uncertainty and suspense arise from different sources, given that uncertainty can exist independently from suspense and, as our results show, audience reports –sometimes a higher– suspense in absence of uncertainty. This is in line with the idea of authors for whom uncertainty is linked to surprise (Smuts, 2008, p. 284) or curiosity (Carroll, 1996b; Wilson et al., 2005; Van Dijk and Zeelenberg, 2007; Bar-Anan et al., 2009; Lowenstein, 2011): both surprise and curiosity –work with but– are not components of suspense. As described in section 2, suspense is described as an effect of anticipation (de Wied, 1995; Wulff, 1996; Allen, 2007), which is independent of uncertainty as an autonomous component of the process of literary reading (Miall, 1995; Guidry, 2004).

For our purposes of modeling stories, we consider this second perspective. According to this, uncertainty can be found without suspense^8^ and suspense without uncertainty^9^ being independent events, it seems more practical to model both concepts as different elements. Curiosity and anticipation may work together, but they are distinct cognitive processes (Beswick and Tallmadge, 1971; McRobert et al., 2011). The fact that the audience is aware of the ending does not in any way restrict the feelings of sadness that a film such as *Million Dollar Baby* evokes when seen for a second time. Actually, the fact that the audience can anticipate the sad ending may make the feeling of sadness even more prevalent. Likewise, spectators feel a vicarious fear by anticipation in a suspenseful Hitchcock scene, and this feeling comes although they have already been warned or previously watched the ending. Both uncertainty and suspense influence the narrative, but they do it in distinct ways. As we propose in our main hypothesis –see section 1–, emotions evoked in the audience involve –although they are not limited to– those coming from curiosity and those coming from anticipation, which are different and compatible emotions.

In this respect and paying attention to the result of our experiment, we cannot support including uncertainty as a component of suspense through adopting the idea of Gerrig and Bernardo (1994, p. 171, 172) that over repeated expositions memory processes provoke an *expectation of uniqueness* –what he calls “anomalous suspense–,” explained as a kind of “systematic failure of memory processes to produce relevant knowledge” (Gerrig, 1997, p. 172) –or “functional amnesia–.” In addition to the refutations already described in section 2, in our experiment, both readings of the same story are consecutive and immediate, which makes it difficult to justify potential memory gaps with respect to the outcome for all the subjects. It does not enable us to discard the role of the memory, but, in any case, these processes do not explain uncertainty as an essential part of suspense. On the contrary, Gerrig's theory acts as an explanation of repeated suspense supposing uncertainty as inherent.

Either way, uncertainty has an impact on the emotions evoked by a suspenseful story. Actually, we agree with the affirmation of Gerrig and Yanal that the audience feels different emotional responses in repeated experiences. As described, Gerrig considers this an “anomalous suspense” (Gerrig and Bernardo, 1994, p. 168), and Yanal (1996, p. 157) defends that the scene does not evoke “the same as feeling suspense.” Both try to explain this fact –derived, in our view, from an inaccurate approach in mixing both concepts (curiosity and anticipation)– in terms of memory failures, or directly differentiating anticipation from suspense. As detailed in section 2, other authors have refuted and exposed the inconsistencies of both approaches. We think the problem arises from the indiscriminate amalgamation of the concepts of curiosity and anticipation under the single label of “suspense” –which, in our view, may be observed in other authors' definitions–. This conflation may be found in the evaluations of the readers, who seem to report as suspense all the emotional spectrum felt during the reading process. Once this response is analyzed in terms of the two separate concepts of curiosity and anticipation, it becomes clear that the evoked feeling is different in the second experience due to the fact that emotions induced by the uncertainty about the outcome are no longer present, but anticipation as a trigger of suspense remains.

We consider that the final emotion changes not because of a memory failure or because it is not real suspense anymore, but because curiosity is no longer playing a role in processing the experience. Consequently, the emotional responses derived from the feeling of uncertainty experienced during the initial exposure to the story are no longer playing an influential role.

In contrast, aspects derived or re-constructed by the certainty of the result –such as empathy or sympathy^10^ with the characters, arising from the knowledge of the outcome now available– come into play. Like uncertainty, they are different from anticipation and interact with it to modulate the emotional effect. In this way, the second time we experience the same story, the emotions coming from curiosity about the resolution of events do not arise, while those coming from anticipation change and still remain.

Actually, observation (c) points toward an interesting possibility: having prior knowledge about the outcome lead to increased perception of suspense in the cases where the story was short. An explanation might be related that subjects might have carefully read the long story the first time and less carefully –less concentrated– the second time; maybe they found the task boring, which affects the immersion in the story. Other explanation is supported by the affirmation of Iwata (2009, p. 174), who comes to define “uncertainty” as “delay in showing the resolution.” Both possibilities merit be contrasted in further analysis of the role of the anticipation with respect to well-known secured outcomes and its implications in a more emotional intensity when the pace of description of events is faster.

Moreover, we account that the use of physiological measures to record autonomic nervous system –i.e., skin conductance– would help to analyze a complementary dimension in the effect of the re-reading. Even taking into account the difficult of identifying when a physiological response is specifically triggered by anticipation, the lack of these measures is a potential limitation of this study, and it must be consider in future experiments.

## 6. Conclusions

This work is based on the hypothesis that uncertainty is not a feature required to experience suspense. Actually, uncertainty –linked to curiosity– and suspense –linked to anticipation– may exist independently. Hence, although we may not deny that uncertainty influences the audience's emotional response to a suspenseful scene, we postulate that this occurs due to the intervention of curiosity, which must be considered as an additional different cognitive process that influences the generation of the final emotion.

To support this hypothesis, we carried out an experiment in which the audience's reactions to uncertainty were evaluated. Results confirm that uncertainty affects the readers' emotional response, but it is not essential for suspense. Moreover, uncertainty does not guarantee a higher suspense than the one that can be observed when the audience knows the outcome.

Additionally, from the point of view of creating a computational model for automatic storytelling systems, separating both types of emotional aspects –uncertainty and suspense– allows us to propose a design in which the complexity of quantifying uncertainty can be replaced by managing what information to provide to the audience. Strategies to achieve this can already be found in the literature (de Wied, 1995; Szilas, 2002; Bizzochi, 2007; Bae and Young, 2008; Graesser and D'Mello, 2012; Lu et al., 2012). This knowledge affects the propagation of the input factors of suspense. This is a promising scheme that must be still refined as part of our proposed system, as explained in section 1.

To conclude, our position proposes a solution for the paradox of suspense, as it denies the basic underlying premise that uncertainty is a fundamental requirement for the perception of suspense.

Regarding other features that are involved in suspense, some open questions remain —for instance, the way in which factors as victim concern are influenced by the knowledge of the outcome—, but we reckon they are beyond the scope of this work. With the objective to enrich our scheme with them, this and other complementary aspects will be studied in a further contribution.

## Ethics statement

The present study is exempt from ethics approval due to the complete lack of risk to the participant's mental and physical health. Nevertheless, all participants were appropriately informed and they explicitly consented to the study and to the data gathering.

## Author contributions

PD, CL, AS, MP-D, and PG contributed to the conception of the study. PD investigated and sorted the related work and performed the analysis. PD and AS designed the experimental method, conducted the experiment and collected the data. PD and CL wrote the manuscript. MP-D and PG provided the critical feedback. CL, MP-D, and PG approved the version to be published.

### Conflict of interest statement

The authors declare that the research was conducted in the absence of any commercial or financial relationships that could be construed as a potential conflict of interest.

## Footnotes

^1^For instance, suspense is used in the area of education as a way to create emotions that can stimulate affective content, influencing positively the memory (Burton et al., 2004, p. 223), and the assimilation of information (Delatorre and Arfè, 2015). On the other hand, in terms of psychological treatment, anticipating events as part of the experience of suspense helps to counteract negative and stressful effects (Zachos and Maiden, 2013, p. 48). Likewise, an adequate management of suspense in video-games directly affect the amusement of either players and spectators (Delatorre et al., in press).

^2^Considering this maximization when there is a 50/50 chance of seeing a preferred outcome (Madrigal et al., 2011, p. 261).

^3^“Readers feel suspense when led to believe that the quantity or quality of path through the hero's problem space has become diminished” (Gerrig and Bernardo, 1994, p. 460).

^4^Based on the work of Van Der Hoek and Lomuscio (2003), an agent is uncertain about a proposition when the agent has to make inferences, leading to the possibilities of being true and false.

^5^Several authors support weakness and innocence as optimal characteristic for ideal victims (Christie, 1986; Walklate, 2006; Smolej, 2010; Fairfax, 2014).

^6^Rafael Marín has published twenty-eight works among novels, anthologies and essays from 1984. It includes mystery and suspense books in Spanish as *Elemental, querido Chaplin: una novela de Sherlock Holmes* (Marín, 2005) or *Los espejos turbios* (Marín, 2012).

^7^Impact of ending knowledge has been included only for the first read, while at second read ending is already known. Likewise, the table does not include more combinations, because they were not significant.

^8^As Smuts (2008, p. 289) affirms: “most of the events in our daily lives are uncertain, but almost none are suspenseful.”

^9^With the only opposition of Yanal (1996, p. 157), who affirms that in repeated expositions the audience does not feel suspense, but “a certain quality perhaps easily misidentified as suspense, namely anticipation.” This position has already been refuted, as explained in section 2.

^10^Aspects not explicitly address by Gerrig and Bernardo (O'Neill, 2013, p. 14).
