# Supplementary material for: Confronting a Paradox: A New Perspective of the Impact of Uncertainty in Suspense
Source: Front Psychol. 2018 Aug 8;9:1392. doi: 10.3389/fpsyg.2018.01392 (PMC6092602; doi:10.3389/fpsyg.2018.01392)
Supplement: Supplementary file 1 [file Data_Sheet_1.pdf]

Story

The story used for the experiment[1] is detailed below. When two columns are presented, the left column presents the good version, in which the main character survives, and the right column presents the bad version. Additionally, in the first phase of each story there is a double column paragraph in brackets, which means that these lines are not presented in the case of uncertain. The story has been translated into English to be included in this paper.

Journalistic style version

1

What you are about to read is the story of a real event that took place at the UCSF Benioff Children's Hospital, in San Francisco, California, on 24 February 2008.

On that day, since 8 a.m., an eight year old boy called Robert Bent and the entire medical team treating him were all ready for his imminent liver transplant. Just the day before a suitable donor had been found, and they were now awaiting the arrival of the organ. However, they were not sure if Robert would survive the wait as his situation was critical.

[Finally, at 21:26, the medical team verified that Robert Bent's newly transplanted liver was functioning correctly, and had not been affected by the damage that it sustained in transit.]

This is the story of what happened.

[Finally, at 21:26, the medical team certified the death of Robert Bent, without having been able to carry out the liver transplant due to the damage that the organ sustained in transit.]

This is the story of what happened.

2

At 08:57 the helicopter carrying the organ landed punctually on the roof of the hospital where Robert Bent was in a critical but stable condition.

3

Two men descended from the helicopter, one of whom was carrying a small blue fridge, in the shape of a case, which stored the fully functioning liver.

4

Minutes earlier, the cleaner had finished mopping the floor of the service stairwell, leaving without displaying the "wet floor" sign.

5

The two men transporting the liver left the roof via the doorway to the service stairwell, which they decided to walk down.

[1] See Delatorre et al. (2018). Confronting a paradox: a new perspective of the impact of uncertainty in suspense. *Frontiers in Psychology* 9. doi=10.3389/fpsyg.2018.01392.

6

As a result, the moment the man carrying the case placed his foot on the steps he slipped, and the case plunged down the stairs.

7

His colleague immediately went to warn the doctors whilst he, after regaining his footing, stayed supervising the case, which was not handled in any way until the doctors arrived.

8

The doctors arrived promptly.

9

When they opened the case, they discovered that the interior bag had ruptured.

10

The doctors took the case to the hepatic laboratory, where the surgeon responsible carried out a biopsy to study the condition of the organ.

11

The analysis showed that it had withstood the impact and it was possible to use the organ for the transplant.

The analysis showed that it had not withstood the impact and it was impossible to use the organ for the transplant.

12

Finally, at 21:26, the medical team verified that Robert Bent's newly transplanted liver was functioning correctly, and had not been affected by the damage that it sustained in transit.

Finally, at 21:26, the medical team certified the death of Robert Bent, without having been able to carry out the liver transplant due to the damage that the organ sustained in transit.

Short novel style version

1

What you are about to read is the story of a real event that took place at the UCSF Benioff Children's Hospital, in San Francisco, California, on 24 February 2008.

That day, since 08:00, an eight year old boy called Robert Bent and the entire medical team treating him were all ready for his imminent liver transplant. Just the day before a suitable donor had been found, and they were now awaiting the arrival of the organ. However, they were not sure if Robert would survive the wait as his situation was critical.

[Finally, at 21:26, the medical team verified that Robert Bent's newly transplanted liver was functioning correctly, and had not been affected by the damage that it sustained in transit.]

This is the story of what happened.

[Finally, at 21:26, the medical team certified the death of Robert Bent, without having been able to carry out the liver transplant due to the damage that the organ sustained in transit.]

This is the story of what happened.

2

At 08:57 the helicopter carrying the organ landed punctually on the roof of the hospital where Robert Bent was in a critical but stable condition.

3

Whilst the rotors were still turning, two men descended from the helicopter, heads down and hair blown by the whirlwind of the spinning blades. One of them was clutching a blue case that was storing, at a temperature of four degrees centigrade and fully functioning, the compatible liver. The other shouted something inaudible. Realizing that he hadn't managed to make himself heard over the noise of the blades, he touched his colleague on the shoulder, pointed at the watch on his wrist and then at the doorway off the roof. The man carrying the case gave a visible nod and, still ducking down, moved away from the helicopter towards the exit. During the journey they had been told that the patient was in a critical situation and they had no time to lose.

4

Just a few minutes earlier, the cleaner had finished mopping the floor of the service stairwell. Like every morning, he had started from the ground floor, working his way up each of the hospital's eight floors. It wasn't a particularly interesting job. The service stairwell had no windows and the only spot of colour on the dull walls was the red of the fire extinguishers, which were always positioned in the same place on the landings between every second floor. Cleaning the corridors was much more enjoyable: the doctors were usually friendly, and there was always a bored patient happy to have a chat. Overall, it was an agreeable job. However, the service stairwell wasn't dirty: the staff used the lifts or any of the three staircases distributed in the building's wings, so there was never anything needing a thorough clean. He whistled as he worked his way up the stairs, with just a mop, a bucket and a cloth hanging from his pocket, and, so as not to have to carry them, he didn't bother to position the "wet floor" warning signs

at the top of each section. That morning, like every morning, he finished mopping in fifteen minutes. He took the lift back down to the ground floor, collected the cleaning trolley and continued his typical working day.

5

The helipad exit was a metal ramp, now under cover, which led to the service stairwell. On the service stairwell landing there was another door leading onto one of the hospital's secondary corridors. This corridor housed the central archives, the main storeroom and, finally, the rooms reserved for doctors and residents. Round a bend at the end of the corridor was the east wing lift.

The man carrying the case went to enter the corridor, but the other caught his arm.

"The patient is on the 6th floor. It's not worth taking the lift," he said, realising his colleague's intention. "By the time we get there and wait for it to come, it'll have taken longer than walking down two floors."

His colleague shrugged and moved away from the door, holding the bannister of the service stairwell with his free hand.

6

His foot slipped the moment he placed it on the first step, twisting his ankle. Since his other foot was already raised he couldn't stay upright. Without thinking, to stop himself from falling he reached out to grab hold of the bannister with his other hand, letting go of the case. The case rolled down the stairs whilst the man managed to hold on and recover his balance. The two men held their breath as they watched in horror how the case spun over on every step, making a noise that sounded like a bunch of loose keys being shaken in a bag.

7

Finally, the case reached the bottom and stopped. Three seconds later the two men were kneeling down beside it, looking at each other. The man who seconds before had been in charge of the case reached out his arm to pick it up again.

"Don't!" warned his colleague, "We don't know if it's been damaged. It's best to call the doctors."

The other nodded, pulling back his hand.

"I'll go," continued the first. "You stay here watching it, in case anyone comes."

Without wasting any time he set off down the stairs, taking care not to slip as well. He went down the two floors and went through the door into the hospital. He walked quickly through the complex network of corridors until reaching the reception desk in the transplant department, from where the medical team was alerted.

8

Meanwhile, keeping a firm hold on the bannister, his colleague hadn't moved. Standing beside the case, he was trying to think of anything except the possibility that the content of the package had been damaged by the fall. His ankle hurt, he was bearing his weight on his injured foot, whilst the other was hardly touching the step. Despite the pain, he had no intention of moving, overwhelmed by the superstition that the condition of that liver in some way depended on the suffering he was capable of offering in exchange.

However, the doctors were soon there. He heard them rushing up the service stairwell. Directly, three doctors appeared, followed by his colleague.

"Move away, please," asked one of the doctors, who was kneeling down next to the case.

9

He heard a click when the doctor pressed either end of the handle with his thumbs. It divided in two, the outer case opening to reveal a sort of small padded fridge. After a brief look the doctor turned to those watching, shaking his head. He closed the case. The fridge had ruptured with the impact, and there was a long split down one side from which a thin stream of refrigerated air was slowly escaping.

10

According to the initial diagnosis, the interior polyurethane bag was urgently transferred to the hepatology laboratory, where the liver was removed for examination. The superficial condition of the organ appeared to be correct. The enzyme activity was still sufficiently low, which gave the medical team hope. Everything depended on the metabolic rate. To check this it was necessary to make a clean puncture and carry out a subsequent biopsy.

With his face covered by a mask, everyone else in the laboratory moved back to give the surgeon in charge space to work. His gloved hands guided a small syringe, the point located approximately halfway down the left lobe of the liver. He felt decidedly nervous. He had carried out numerous biopsies but never before had a child's life been hanging in the balance. If he was one millimetre out he would damage the organ irreparably.

11

Holding his breath, he inserted the needle one centimetre. Relieved to not encounter any resistance, he carefully drew out the plunger. One quarter of the syringe filled with a transparent liquid, which he passed to one of his colleagues. Satisfied, he wiped the sweat from his brow with his uniform and watched as his colleague put barely two drops of liquid on a Petri dish and placed it under the microscope. The surgeon lowered his mask and looked down the tube. As he analysed the sample, he sporadically pursed his lips, occasionally lifting his head to blink several times.

After an interminable half a minute, he moved away from the microscope and looked with concern at his colleagues, who were anxiously awaiting the diagnosis. The metabolic rate gave cause for optimism: the organ had not been damaged by the impact.

Holding his breath, he inserted the needle one centimetre. Relieved to not encounter any resistance, he carefully drew out the plunger. One quarter of the syringe filled with a transparent liquid, which he passed to one of his colleagues. Satisfied, he wiped the sweat from his brow with his uniform and watched as his colleague put barely two drops of liquid on a Petri dish and placed it under the microscope. The surgeon lowered his mask and looked down the tube. As he analysed the sample, he sporadically pursed his lips, occasionally lifting his head to blink several times.

After an interminable half a minute, he moved away from the microscope and looked with concern at his colleagues, who were anxiously awaiting the diagnosis. The metabolic rate confirmed his worst fears: the organ had definitively deteriorated as a result of the impact.

12

Finally, at 21:26, the medical team verified that Robert Bent's newly transplanted liver was functioning correctly, and had not been affected by the damage that it sustained in transit.

Finally, at 21:26, the medical team certified the death of Robert Bent, without having been able to carry out the liver transplant due to the damage that the organ sustained in transit.
